# Pathological Investigations

**Published:** 1840-04

**Authors:** 


### ART. V.

*Pathologische Untersuchungen.* Von Dr. HENLE, Prosector und Privatdocenten in Berlin.—*Berlin*, 1840. 8vo, pp. 274.
*Pathological Investigations.* By Dr. HENLE.—*Berlin*, 1840.

WE are rarely sanguine of finding much that is profitable in German works on Pathology; but if there be a writer in that country from whom more than from any other we should anticipate solid excellence in whatever subject he undertakes, it is Dr. Henle, the laborious and clever assistant of Professor Müller. Though young, his reputation in anatomical science is already among the highest in Europe, and the character of his annual account of the progress of pathology, in Müller's *Archiv*, is sufficient proof of his learning, if not of his knowledge in that department.

The work before us is the first of Dr. Henle's that we have seen in which excellence is alloyed. With much that exhibits an extensive theoretical knowledge of medicine, it contains much more that affords evidence of ignorance of its practice. But we will give, in a connected form, the substance of the first two and best of the four papers, whose subjects are Contagion and Sympathy.

I. *On Miasmata and Contagions, and on Miasmo-contagious Diseases.* The object of this paper is to establish a probable, if not certain, theory, that the material of contagion is composed of organic matter, and "not only an organic but a living matter, endowed with individual life, which stands to the diseased body in the relation of a parasitic organic being." (p. 15.) The chief grounds for this view are drawn from the phenomena of certain contagious diseases attacking the lower animals, especially those of the muscardine in the silkworm; and, from the connexion lately proved to exist between fermentation and the development and increase of infusoria and the minute algæ. With these as facts, and with some ingenious reasonings, the author endeavours to show how all the phenomena of those diseases, which he presumes commence from miasma and spread by contagion, including smallpox, measles, scarlatina, dysentery, cholera, typhus, plague, certain forms of coryza and catarrh, one kind of puerperal fever, and some others, may be explained by his theory.

For the organic nature (whether independently living or not) of the matter of contagion, the chief arguments he advances are these: 1st, The same epidemic will rage under all circumstances of atmospheric and other physical external conditions; and we are, therefore, obliged to assume for its cause some poisonous material mixed in the air, and capable of being carried about in it and separated from it. 2d, The organic nature of this material is rendered probable, by its power of increasing by the assimilation of foreign substances, a power which, so far as we know, is peculiar to organic beings. Fermentation is, indeed, often advanced as a proof of the contrary; but the observations of Cagniard-Latour and Schwann prove that it is itself only the decomposition of organic fluids effected by the agency of vegetables of the lowest class, which grow and increase at the expense of the azotic and saccharine materials in the fermenting liquid; and hence the analogies between the mode of development of an infectious material and fermentation are stronger arguments for than against the organic and vital nature of the former. Thus, 3dly, the action of contagion is analogous to fermentation in that the quantity of the effect produced bears no relation to the quantity of the ferment employed; a circumstance depending, in the propagation of contagion as it does in fermentation and putrefaction, on the prolific power of the organic beings in each. 4th, The accurately typical course of the miasmo-contagious diseases is evidence of a periodical development, such as occurs only in organic beings. The whole course of these diseases is marked by peculiarities in regard to *time*, such as never occur in diseases from common causes, and such as show that the cause of the disease has itself a regular course of periodical development; a peculiarity known only in living beings. The continuance of an individual case may perhaps be compared with that of one generation of parasites; the continuance of an epidemic with that of a genus. One observes in organic mixtures that certain genera of infusoria appear, exist for a time, and then vanish and make way for new ones; one genus seeming to serve for the generation, or rather for the nourishment, of another; and in this there is a striking analogy to many epidemic diseases, which, having affected the body once, leave it for the rest of the time that they may last in the neighbourhood, or for the rest of life.

The matter of miasma (that is, of that which, generated out of the living body, produces a certain disease when applied to it,) is evidently identical with that of contagion, or of that which is separated from the living body that has been attacked by the miasma. "Contagion is, as it were, miasma in the second generation; a miasma which has passed through the first epoch of development within the living body." ....They may both be called *matter of infection;* and this "is constantly the same for each specific disease: it appears as contagion when its origin can be directly traced to a diseased body, and as miasma in the opposite case." The phenomena of the production of the matter of infection in diseases which are not contagious, are deducible as analogies to the same phenomena in contagion; and among the former, there are found, as evidence of the vitality of the poison in both, 1st, That miasmatic diseases, as agues, are endemic where there is a constant decomposition of organic substances going on, as near bogs, after floods, on board ships, &c.; but this decomposition is, in fact, nothing else than the change of organic matter produced by infusoria and the minute algæ; so that every putrefying body is, as it were, a breeding-place of these living beings; and where organic matter is thus exposed widely, and in great quantity, the whole atmosphere must be filled with their germs; 2d, That the same means which favour, or limit, or destroy the formation of these lowest living beings, also favour, limit, and destroy the action of infecting matter, as acetic acid, whose beneficial influence in hospital-gangrene is well known. Warmth and moisture also favour the development of both; oxygen is essential to the life of all organic beings, and miasmata are destroyed in irrespirable air; but the ova of infusoria, &c., may remain alive without oxygen, and so also contagion sometimes remains latent for years, without increasing or developing itself. In like manner, both infusoria and parasitic vegetables will remain dry for years, and yet be capable of revival; and so will the matter of contagion. For the difference between merely miasmatic and miasmo-contagious diseases, it may be assumed, that in the latter the contagion can produce germs in the diseased body, in the former not.

But the theory is not all drawn from such analogies; one contagious disease, at least, is known to depend on the development of a vegetable parasite—the muscardine of the silkworm.* The characteristic signs of this disease first appear after the death of the worm, whose body becomes covered with a white powdery efflorescence, and then dries and mummifies. This efflorescence is a minute vegetable, according to Montagne, a species of botrytis, by the contact or inoculation of which the disease is communicated. The germs of this plant increase by the consumption of the body in which they are placed; after the death of the worm, they perforate its skin, and grow up the more, the warmer, moister, and more quiet the surrounding atmosphere is. They then gradually dry, and form a light powder containing their germs, which, on the least motion of the worm's body, is scattered in the air. The germs are so light and so numerous on a single individual, that they spread about with the greatest rapidity, adhering to bodies of all kinds, and remaining long suspended in the air. They retain their contagious power for as much as three years; and the ova of silkworms affected by the disease may communicate it to healthy worms, by the germs of the parasite that adhere to them. In how many thousand other ways the disease may be carried cannot be imagined. It is most severe in the spring, and its spreading is favoured by warm and dry weather; it chiefly attacks well-nourished worms, and the common disinfecting means are the best for putting a stop to it. The tissue in which the parasitic plants chiefly grow, is the pigment beneath the skin and the subcutaneous adipose substance; they spread from the point of inoculation by root-like growths, and by single globules separating and floating in the adjacent fluids, and perhaps passing into the blood.

It must be admitted that, in all these essential points, the muscardine is analogous to the miasmo-contagious diseases of the higher animals.

"Under favorable circumstances (in its case in mouldy moss) the cause of the disease forms independently, as miasma; with heat and dryness it becomes epidemic and contagious, and spreads further only by contagion. On its decrease, the epidemic diminishes, and its contagiousness ceases. Currents of air convey the contagion to great distances, so that the disease may break out at any place, as if from a fresh miasmatic origin. The contagion is thus at once aeriform and fixed, so that it may be inoculated; it is destroyed by the common disinfectants; it retains its power, when dry, for years; an imponderably and immeasurably small quantity of it is sufficient to develope the disease into the most virulent epidemic. The most vigorous worms suffer most, and form most fresh matter of contagion. Chemical remedies are useless, either for the prevention or cure of the disease." (p. 40.)

Nor is this the only disease of the kind that occurs in the lower animals. Ehrenberg has discovered a vegetation growing on fish, and

* The most important facts relating to this subject may be read in the Annales de Sciences Naturelles, tom. viii., ix.

generating disease in them. Henle has found vorticellæ growing on the toes of tritons kept in a glass vessel, destroying and producing a kind of gangrene of the parts (chiefly between the toes) to which they are attached. And Hannover has made exactly similar observations on another species of triton, and proved that the mould growing on them is capable of inoculation, and is distinctly contagious.*

It is admitted, that "we look round in vain, through the whole range of epidemic diseases in men and the higher animals, for an observation parallel to the above, to prove the similarity of a contagious matter to known animal or vegetable bodies." (p. 41.) But the difficulties of obtaining such observations are many, evident, and perhaps insuperable; so that the absence of such facts is not sufficient to negative the deductions from analogy and reasoning; and the theory may fairly claim reception, if it will stand the test of being applied in the explanation of the phenomena produced by a contagion operating in the body. "How, then, on the supposition of a *contagium animatum*, can the symptoms and course of miasmo-contagious diseases be explained?" (p. 20.)

The parasitic beings which constitute the matter of infection may be considered as belonging to the lowest and most minute, yet the most prolific, of creatures. By their action on the living body, inflammation or putrid decomposition is produced. Now, putrefaction is, in fact, not a mere decomposition of organic matter into its elements, but an alteration of it, effected by infusoria, as the vinous fermentation is a change of organic matter by the action of minute vegetables; and an evident formation of such parasites is often observed on the bodies of living insects, and even birds. Hence the supposition of parasitic beings as the material of contagion will hold in the cases of epidemics with putrid decomposition, as hospital gangrene.

"In general, the individual power of an organized part has a stronger affinity for the nutritive materials of the body than a foreign organization has; but that the latter may gain the ascendancy, is proved by the formation of entozoa. One may say, that while in common putrefaction the infusoria decompose organic matter already dead, in these cases it is at once killed and decomposed by the contagious parasites." (p. 22.)

In the acute miasmo-contagious diseases with inflammation of the membranes, and especially in the contagious exanthemata, as they are called, the disease may be supposed to commence with the reception of the parasites or their germs. They are received from without only on mucous membranes or injured parts of the skin, and often, on particular spots, varying according to the nature of the parasite, just as particular parts are liable to be infested by particular entozoa. It is remarkable that the mucous inflammations often occur without any cutaneous eruptions, but the latter are always preceded by the former, unless the disease be communicated by direct inoculation, and then the former are often altogether wanting.

In the first period of the disease, the parasites produce no evident symptoms; they either remain as yet undeveloped, or it requires some

* These observations, which were made subsequently to the writing of his book, are alluded to in the author's preface.

time for them to increase enough to have a sensible effect. In the next period, inflammation or putrefaction takes place; the latter (as in hospital gangrene, angina maligna, &c.) resulting, on the one hand, from the nature of the contagion, on the other, from the depressed constitution of the patient; the quantity of contagion has probably no material influence. (Many of the early symptoms are ascribed to reflex actions from the spinal cord, as the sneezing in measles, the cramps and spasms, the shivering, &c., which are produced according to the laws of sympathy, explained in the second paper.)

The inflammation which the contagion produces is more or less superficial, whether it affect the skin or mucous membrane. Its passage from the spot it first attacks may take place in three ways:

1st. By the parasites, as they increase, extending on the body, and passing along or under the cuticle. In the latter way, they may travel, as the inflammation usually does, from the mucous membranes to the skin, or, as it does more rarely, in the opposite direction. Perhaps the parasites travel especially towards the skin for freer access to the oxygen; and it is remarkable that all acute exanthemata which begin in the mucous membranes of the nose, mouth, and eyes, spread from the head towards the trunk; while in dysentery, of which the contagion acts chiefly on the lower bowels, the eruption when it breaks out generally commences on the abdomen. It would argue strongly for this view, if, after inoculation, the eruption always spread from the point of insertion; but this is not certain, though there are some facts in favour of it.

2dly. The inflammation may spread by sympathetic irritation of parts of the skin which the parasites have not yet reached, as in the itch the eruption spreads to parts in which no acari can be found. But this explanation will not hold for the cases in which every inflamed part of the skin contains contagion, as in smallpox.

3dly. The inflammation may spread through the medium of the blood, but this (though it cannot be fully disproved) is considered as highly improbable.

The fever and the other general symptoms may be referred to a twofold source: 1st, They may be considered consequences of the local inflammation, as in non-miasmatic inflammations; or, 2dly, as the result of the alteration of the blood or the organic substance on which the parasites have fed and increased. The blood must be altered, just as a fermenting liquid is, by the decomposition which the infusoria produces in it; and in many cases the blood is evidently changed both in physical and chemical properties. This alteration is probably the most influential cause of the fever; for it is more intense than the fever which arises from a common membranous inflammation of the same extent, and unless it is produced, the disease is not fully developed, nor is the patient secured against a second attack.

The disease ends favorably when the parasites cease to live or to grow, perhaps after they have produced contagious germs. The pustules and vesicles which may be formed (and which must be regarded not as producers of contagion, but as simple cutaneous inflammations, producing common pus, according to common pathological laws) open and discharge their fluids impregnated with contagious germs, or they dry up, and their scales, containing dry contagion, fall off. In this manner, and in the desquamation of the skin, as in measles and scarlatina, the contagion must become mixed as dust in the atmosphere around the patient; or, if the exanthema were seated in the lungs or intestines, then the sputa or the excrements are impregnated with the products of the inflammation and the germs of the parasites contained in them, and thus with the close of the inflammation the disease may be terminated.

On the other hand, the fever and the other accidents which ensue on the action of the contagion, may continue after the latter has ceased to have any effect as a cause of them; they may continue as a common fever, or common suppuration, &c., and may produce a long convalescence, or death.

The metastases observed in exanthemata (when they are real and not merely the result either of local disease establishing itself in the general depression which the contagion produces, or of some other accidental complication,) may be explained either by the parasites being hindered from developing themselves externally, and therefore spreading more in the opposite direction, or by the loss of equilibrium which ensues when a preponderant excitement of any part is suddenly checked—a phenomenon which will be explained only when all the causes of the sympathetic connexion of parts are discovered.

Such is Dr. Henle's theory of animate contagion. Many of our readers are aware that the same subject was very ably treated by Dr. Holland,* in his Medical Notes and Reflections; and, as far as was possible, by lucid arguments, and by fair deductions from analogies, it was there shown that, for an explanation of the phenomena of cholera (selected as an example of the whole class of epidemics), no hypothesis was open to so few objections as that of minute insects infesting the body, and transmitted from one individual to another, through the medium of the atmosphere. Whoever adds to the arguments of that paper the facts of this, must confess that a strong case is now made out in favour of taking the direction which they indicate in future investigations of epidemics. The case stands simply thus: an analogy has long been observed between the fermentation of organic fluids and the influence of contagions on the blood; fermentation is closely connected with, if not caused by, the influence of parasitic animals and vegetables in the fluids in which it is going on; there is, therefore, a reasonable probability that the matter of contagion may be composed of parasitic beings or their ova; and this probability is strongly supported not only by its hypothesis fitting the phenomena better than any other yet imagined, but still more strongly by the fact of some contagious diseases in the lower animals being visibly the effects of parasites.

The defects of the hypothesis, however, are evident, though not desperate; the chief of them being the distance of the analogy of the diseases of worms or amphibia and of men, and the absence of any actual observation of such parasites as are supposed to constitute contagion in the bodies of the higher animals.

* Dr. Henle does not quote this part of Dr. Holland's work, though he has extracted facts from other portions of it. Probably the paper on contagion was printed before Dr. Holland's work reached Berlin.

The explanation of all the symptoms of contagious diseases, by the direct influence of the parasites, is quite unnecessary; and we think Henle has weakened his case by throwing this burden upon it. If the course of the epidemic, up to the period of its attacking the individual, and its very first symptoms, can be shown to depend on the development of the parasites, enough will be proved; after that time, other causes of symptoms, as the altered state of the blood, and the impression of the nervous system, must at once come into operation, and there are few morbid phenomena which these, with their long train of subordinate influences, are not amply sufficient to produce.

II. *On Nervous Sympathies.* This important and difficult enquiry is the subject of Dr. Henle's second paper. The phenomena are all arranged as illustrations of this proposition: "It is matter of observation, that nerves communicate their excitement one to another; and it is proved that this communication takes place (at least in the case of the animal nerves) only within the central organs. Communication of excitement in the central organs is the foundation of all nervous sympathies. The laws according to which excitement spreads in the central organs, are also the laws of (nervous) sympathy." (p. 83.)

It is impossible to imagine this communication of excitement, except as taking place from one point to another adjacent to it; nor is the distance of the parts that sympathize any hinderance to this view; for it may be that in the central organs nerves are near each other which diverge as they proceed to the periphery. Suppose the origins of two fibres, A and B, to lie near each other in the brain or spinal cord, and that one of these fibres goes to the right and the other to the left side of the body; the stimulus of the portion in which they originate must produce sensation or motion on both sides of the body. If a communication of excitement is effected between two such filaments in the central organ, the stimulus of A extends to B; and since sensitive nerves propagate their excitement centripetally, the effect is the same whether one be stimulated at its peripheral or at its central extremity, and the excitement of the peripheral end of A will, as in the preceding case, be communicated to B, and its influence will appear at its (B's) peripheral extremity; because, in whatever part of its length a nervous filament is stimulated, its excitement is evidenced at its peripheral end.

Were our knowledge of the arrangement of the origins of nerves in the nervous centres complete, sympathies were thus easily explained; but at present all that can safely be assumed is that the origins of the nerves are arranged *nearly* in the same series in which they come out from the brain and spinal cord, and that *in general* the nerves of adjacent parts of the body arise near one another in the central organs.

From a lengthened consideration of the functions of the sympathetic system of nerves, and an interesting comparison of the analogies of cellular tissue and organic muscles, the author concludes as follows:

"1st. The nerves may also communicate their excitability to one another within the ganglia, and thus the ganglia may be considered as in some degree central organs, and as media of sympathies. Since, however, all nerves which are connected together by ganglia do not terminate in them, but are prolonged to the central organs, it is in most cases, when the brain and spinal cord are perfect, impossible to determine whether the communication is effected in one of them or in the ganglia. The ganglia must always, therefore, occupy a subordinate place in the enumeration of the sympathies, unless more be ascribed to them than is warranted by observation, or unless they are made to bear the burden of everything, which nothing else can explain. 2dly. The organic nerves are the motor nerves of the involuntary muscles, and perhaps of the cellular tissue and the vessels, and as such form a subdivision in the class of motor nerves." (p. 106.)

There are three directions in which excitement may be communicated in the spinal cord: 1st, in its breadth, from one nerve or column to the corresponding one on the opposite side; 2d, in its length along the same column to a similar nerve above or below; 3d, in its thickness from one column of one side to the other on the same side. To communication of excitement in one of these three ways, all sympathies may be referred.

1st. Symmetrical communication between the corresponding nerves of the two sides of the body. Instances of this are seen in the pain of a healthy tooth, when its fellow on the opposite side is painful and carious, and in the contraction of both pupils when strong light is applied to only one.

2d. Vertical communication in the same column. Almost every severe pain is felt around the part which is diseased; as that from a tooth over all the face, from a finger up the arm, &c. Strong light produces tickling in the nose; a disagreeable sound produces pain in the teeth and corrugation of the skin. (And so on, through a number of similar examples; but the facts that may be placed in each division of this excellent arrangement of sympathies, the reader must himself supply.)

3d. Antero-posterior communication; to which may be referred all the phenomena of reflex motion, in which motor nerves are excited by communication from the sensitive. Many of these are phenomena of healthy action, as the contractions of the pupils, the pharynx, &c.; but the reflex motions in the voluntary muscles usually occur only in abnormal states, as in narcotism, paralysis, &c. In this class also may be included not only the sympathies in which motions follow sensations, but those also in which increased secretion follows sensation, as in the flow of tears when the nose or eye is irritated, &c.

"What conditions favour the communication of nervous excitement in the central organs, and thereby the production of consensual excitement?" The extent of communication depends on, 1st, The strength of the stimulus; as Mr. Grainger has shown, the extent of reflex motion depends in all cases on the intensity of the stimulus;—2d, on the kind of stimulus; one blow, or prick, or other application of stimulus, does not so easily excite reflex motion as repeated slight irritations, &c.;—3d, on the excitability of the whole system, and the degree of vital power.

These observations relate rather exclusively to normal sympathies; and this portion of the paper presents, on the whole, by far the most rational account of the subject that we remember to have read; every illustration is apt, and every argument judicious; but in the rest, which treats of "Morbid Sympathies," the author is less happy, because less familiar with the facts of his subject.

The sympathies of the nerves, he says, may be anormal in two ways; either when the communication takes place more speedily or in a greater extent than in the healthy state, or when parts act in association which in general do not communicate their excitement to one another.

"a. Anormally increased consensus. 1st. Since communication is the more sure and more extended, the more violent the stimulus; and since, with equal external influences, the effect of the stimulus is the greater, the greater the excitability; therefore increased excitability of the sensitive nerve, which is first stimulated, must favour the diffusion of the excitement." (p. 121.) Thus, for example, in congestion and inflammation a common degree of warmth produces a burning sensation, the skin being in a state of exalted excitement; and again, in inflammations, many of the common reflex actions are greatly increased in intensity, as the violent contraction of the muscles of the eyelids, when an inflamed eye is exposed to the light, the spasmodic contraction of the bladder, when (though the urine is unaltered) the mucous lining is inflamed, &c.

2d. "The communication must be more easy and more extended when the primarily stimulated nerves have their normal excitability or excitement, but the nerves associated with them by contiguity have an exalted excitability," (p. 122;) for the first are to the second in the relation of an external stimulating substance. An instance of this morbid state is found in the impotence that arises from increased irritability of the seminal vesicles, so that after the slightest irritation emission takes place. Hence also, in general irradiation from any point of the nervous system, the associated excitement is especially great in the muscular or sensitive nerves which are already morbidly excited; as patients with diseased lungs cough more from the same slight irritation than healthy persons, &c.

3d. The same effect occurs in certain conditions, not merely of increased excitability of a particular set of nerves, but where the whole nervous system is thus altered. Thus, among healthy individuals, the nerves seem to be more closely connected with each other in women, and in those of a sanguine temperament, than in others; and to this may be referred in disease all the phenomena of nervous excitability, irritable debility, or erethism, &c. In this category may be placed also the over-excitement produced by watching, by narcotics, &c., in which hallucinations and spontaneous cramps occur; here, too, come tetanus, and the condition produced by excessive loss of blood: in all these cases, the increased communication of excitement is only the consequence of the generally increased excitability. Another series of similar phenomena are seen in those sympathies which occur in the reflex motions of paralyzed bodies and during sleep, in which the author thinks that the cramps, contractions, and exalted sympathies do not depend, as Dr. Marshall Hall (in our opinion more correctly) supposes, from the mere want of the influence of the brain, but from an organic alteration of the spinal cord, and commonly from the same as that on which the paralysis depends. A third series of these phenomena of generally increased communication from generally increased excitability, are found in the greater ease and extent of reaction upon the same stimuli, when the mind is intently occupied, as in starting when, being closely occupied in some mental effort, a sudden noise is heard, or a sudden contact felt.

From all these considerations, the author concludes that "there is no peculiar organic disposition to sympathies, but that the diffusion of associations depends only on the strength of the stimulus and the degree of excitability." (p. 133.)

B. Acquired abnormal or individual sympathies, in opposition to the congenital, normal, and general, are those which are established between parts, the origins of whose nerves do not stand in direct anatomical connexion. In these cases it appears as if the associated stimulus, instead of passing from one nerve to certain others, as in common sympathy and in the healthy state, or instead of passing, as in diseased excitability or unusual excitation, beyond its natural limits, or over the whole system, passed as if by leaps from this to that point, missing others; and it seems as if disease, habit, or idiosyncrasy could open new ways for communication in the central organs. These uncommon sympathies are seen when one part of the nervous system is preeminently excitable; and so in a general excitement its affection is most prominent, and it appears as if it were in sympathy with all parts of the organization. This part is what the older pathologists called *pars minoris resistentiæ.* Hence, when a tooth is carious, anything unusual may produce toothach, whether heat or cold, or errors in diet. These sympathies are seen also in the reactions that take place after peculiar affections of the senses. In the impressions on our senses, we feel ourselves excited not only in a greater or less degree, but also in a certain manner, or by a certain quality; this quality of the sensation excites ideas, and these again sensations or motions; and these influences of the mind appear like sympathies following the peculiar impressions.

*Antagonism* is the next subject considered:

"When any part of the skin is stimulated, the reaction, congestion, and inflammation extend over a greater or less portion of the surrounding skin; this reaction is sympathetic. When, therefore, fresh inflammation is excited in the neighbourhood of an inflamed part, the first is sympathetically increased. But if a stimulus producing congestion or inflammation is applied at a certain distance from the affected part, the reaction in the latter is lessened, and this decrease is effected by *antagonism.* Sympathy and antagonism are both consequences of a connexion between the stimulated parts. According to the violence of the stimulus and the excitability, an increased excitement diffuses itself in a definite circuit; but as with respect to time excitability is in general destroyed by stimulus, and the more rapidly the greater the portion of the body stimulated, so also, with respect to space, an exaltation of excitement in one part produces a decrease of it in another. .... And in general, the nearer the antagonist and derivative excitant is applied to the diseased part, the more efficient it is, provided only it is not placed so near that a sympathetic affection may ensue." (p. 138.)

The central organs are in like manner subject to be affected by the connexion of parts, sometimes by sympathetic and sometimes by antagonist excitement, though it is scarcely possible to say under what circumstances each is observed; but in general it is observable that certain parts of the nervous system are so connected, that when the excitement of one is increased, that of the other is also; that in others the excitement of one part sometimes exalts and sometimes lowers that of the other; and that, lastly, under certain circumstances, the connexion of parts is always shown by antagonism.

The evidences of this antagonism are seen in the common processes of counter-irritation and derivation, whether the antagonist phenomena be merely pain, or other simple nervous excitement, or inflammations of adjacent parts; but the applications which Henle makes of the principle of antagonism are shown in the parts which follow his account of the "sympathies of the organic nervous system." (p. 142.)

He believes, from many and some good reasons, that the cellular tissue of the skin and blood-vessels has an energy analogous to that of the muscles, and since this *tone* of the cellular tissue may be altered by various circumstances, he asks, May not such alterations be also induced indirectly by sympathy? May they not depend, like the motions of the muscles, on an altered state of the sensitive, or, to speak more generally, the centripetal nerves? Facts are not wanting to prove a connexion between the sensitive and the organic nerves; the flow of tears on stimulating the nose or conjunctiva, and many others such, are sufficient. And in these it is usual to consider the increased secretion as a sign of increased activity, and it is regarded as the result of sympathy between the organic and the sensitive nerves. But the vessels of the gland, during increased secretion, are probably enlarged and relaxed; and this dilatation can only be the consequence of their decreased excitement; and thus it would appear, that the increased secretion, following the excitement of sensitive nerves, is not sympathetic but antagonistic, an increased excitement of centripetal nerves producing a decreased tone of the cellular tissue. And Henle now sets himself to show how this theory will adapt itself better to the various phenomena of inflammation, &c., than the old one of increased action and exalted tone.

It is in the first place evident, he says, that the nerves of the involuntary muscles are sometimes in an antagonist state to those of the voluntary, as in the tympanites and retention of urine, which often accompany the spasms of the voluntary muscles in hysteria, and in other like cases. If we next consider the cellular tissue under the idea that its contraction corresponds to increased, and its relaxation to decreased excitement of the organic nerves, we find it sometimes in sympathetic, sometimes in antagonistic connexion with the animal nerves. *Sympathetically*, the dartos contracts in tenesmus, the nipples become erect when the skin around them is rubbed, &c. *Antagonistically*, the joints feel loose in the hysterical, goose-skin often coincides with paralysis, &c.

The contraction of the cellular tissue on applying cold, and its relaxation on applying heat, also appear antagonistic, because, for many reasons, we must believe that cold depresses the excitement of the sensitive nerves, and that heat exalts it. In all these changes, to which cellular tissue is liable, the blood-vessels participate by their external cellular and contractile coat; their tone is expressed in the varying turgor of parts; if their turgor is increased, they contract, and secretion diminishes; if it is decreased, they dilate, and redness, swelling, and congestion, with increased secretion, are produced. Now whether the vessels can be sympathetically excited with the animal nerves is uncertain; but that they are antagonistically, is evident from many facts, as in the sweating of exercised parts, the flow of saliva on working the muscles of the mouth, &c.

The cases of consent between the sensitive nerves and the nerves of the vessels, are divisible into two series:

1st. Excitement (antagonistic) of the nerves of the vessels from internal causes, as shown by the increased secretion, œdema, redness, &c., which are often seen coincidently with neuralgia, in which the sensitive nerves, having their excitement exalted, that of the organic nerves is depressed, and the cellular tissue of the vessels is dilated. The same connexion is shown in the ease with which parts, whose sensitive nerves are already highly excited, become inflamed.

2d. Excitement of the sensitive nerves from external, chemical, or mechanical stimuli, in which the antagonism of the organic nerves of the vessels is always shown by the congestion and inflammation that ensue. From this fact, the increased fullness of the vessels that always follows the pain in any part, Henle finds himself, "almost unexpectedly, on the way" to the ultimate object of all of which we have made the preceding abstract, " a theory of inflammation."

" Congestion and inflammation depend for their proximate causes on relaxation of the capillaries, and this is antagonistically produced by stimulus of the centripetal nerves." (p. 152.) It will be sufficient if we present the most favorable illustrations of this theory, those drawn from " the prototype of inflammation, that which is traumatic." Here all the cardinal symptoms result from the primary affection (excitement) of the sensitive nerves; the pain, directly, as it is in fact always the first, and the redness and swelling from the antagonistic paralysis of the vessels. The objective heat also depends, as is now unquestionable, upon the nerves, either directly or through the medium of nutrition. Thus, for inflammation there are two factors, increased excitement of the sensitive nerves, and dilatation of the vessels; and these are produced the one by the other.

Now since, according to this supposition, the cause of all the objective symptoms (the heat excepted) is atony of the vessels, which, in true inflammation, is antagonistic, but which may occur primarily, it is evident that redness, swelling, ulceration, and gangrene, but without pain and heat, must take place whenever the nerves of the capillaries are primarily paralyzed. And that these symptoms do occur, in such circumstances, is clear from Magendie's observations of the sloughing of the cornea after division of the trigeminus, the frequent ulcers, &c., in paralytic limbs, the gangrene in limbs in which the ischiatic nerve has been divided, &c.

As we have already said, the first part of this paper affords the best arrangement we have yet read of the various nervous sympathies, and clearly indicates the laws to which a great number of the bizarre collection of facts, once heaped together under that name, are referable. But as the author approaches more nearly to his theory of inflammation, his excellence rapidly diminishes. To the theory itself there appear two chief objections; that it is founded on too many assumptions, and opposed to too many facts. Thus, there are assumed for it—the probability that the coats of capillaries have a tone or contractility; that this is governed by the organic motor nerves; that these are capable of antagonistic association (itself a relation, which is in any case only probable) with centripetal nerves; that the result of their depressed excitement would be dilatation of the capillaries; and that the result of this would be increased secretion or inflammation. Now, even if we granted a fair share of probability to each of these assumptions, it is yet certain, from the common and calculable laws of probable events, that the chances are at least 1000 to 1 against the hypothesis composed from them all, containing the expression of the true proximate cause of inflammation. And against this small chance many facts may be set; as (to mention but a few), that if this antagonism followed the common laws of nervous association, the intensity of inflammations should bear a direct proportion to the intensity of pain precedent or coincident, whereas many diseases have great pain without inflammation, and many inflammation without pain, and in none is there any constant relation between the two; that, again, persons or parts with the least tone of cellular tissue should be subject to the most severe inflammation; that, were this hypothesis true, local bleedings should do harm by increasing the excitement of centripetal, and decreasing that of motor nerves, and so on.

The author does not recover himself in the rest of the work. The other papers, "*On the Course and Periodicity of Disease,*" and "*On Fever,*" are most strange productions. Choosing for consideration only those subjects which are least illustrated by facts, the author raises difficulties hitherto unimagined, as if only to make confusion of their ruins when he has thrown them down again; but we threaded the maze of words in vain to find one explanation of subjects which, though always obscure, never before seemed so densely dark.

It is singular, that a person who, like Dr. Henle, is so well acquainted with the difficulties of investigating one branch of science, should imagine that all the uncertainties in another far more difficult branch, may be disposed of by endeavouring to adapt a plausible theory to ideas which have as yet scarcely the semblance of facts. No one is more cautious than he of receiving, as *fact*, a microscopic observation; and yet he gives credence to assertions in medicine which are scarcely more certain than matters of popular belief. As an *observer*, however, these papers do him no discredit; he has only committed the errors of believing it possible to write correctly on medicine without personal observation, and of building loftier theories on loose assertions, than even the solid truths of demonstrative science could safely bear.

